# Supplementary material for: Reminders of past choices bias decisions for reward in humans
Source: Nat Commun. 2017 Jun 27;8:15958. doi: 10.1038/ncomms15958 (PMC5490260; doi:10.1038/ncomms15958)
Supplement: Supplementary Information [file ncomms15958-s1.pdf]

Title of file for HTML: Supplementary Information
Description: Supplementary Figures, Supplementary Tables, Supplementary Notes and Supplementary Reference

Supplemental Figures

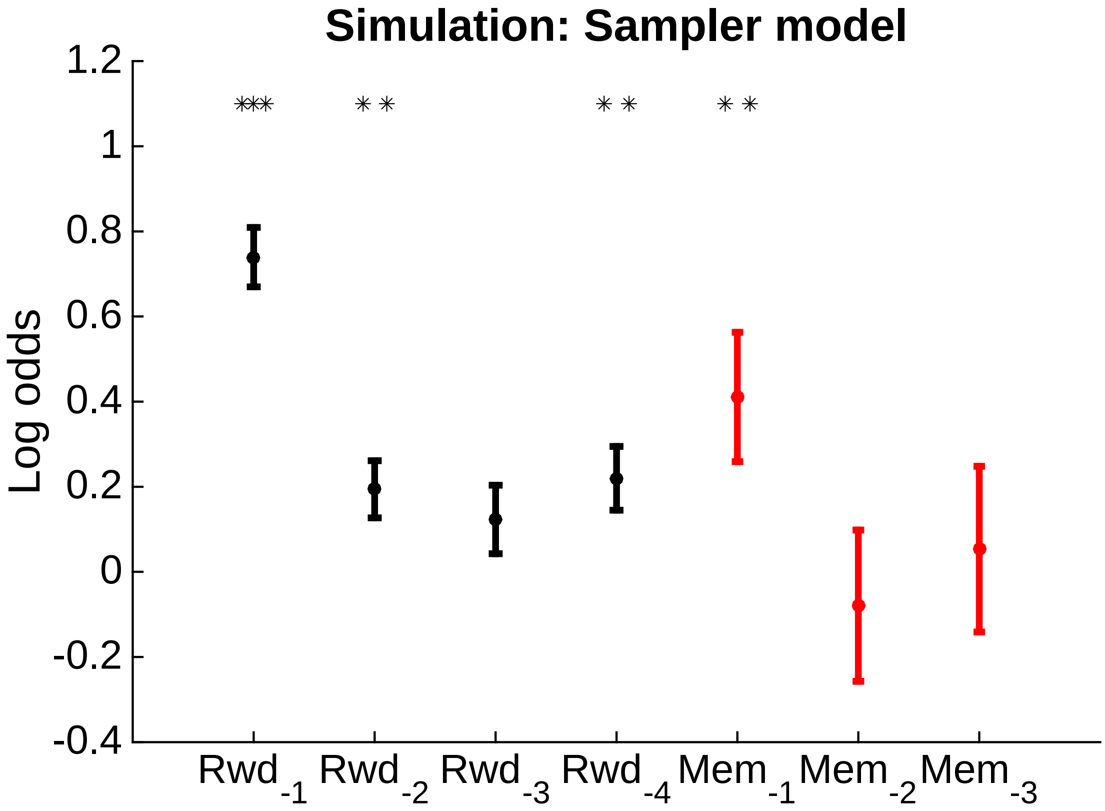

Figure S1: **Regression analysis on simulated subjects.** Average regression results for 50 populations of 20 simulated subjects each. Subjects were simulated using the Sampler model at the parameters fit to the study population. Error bars are SEM across the population means.

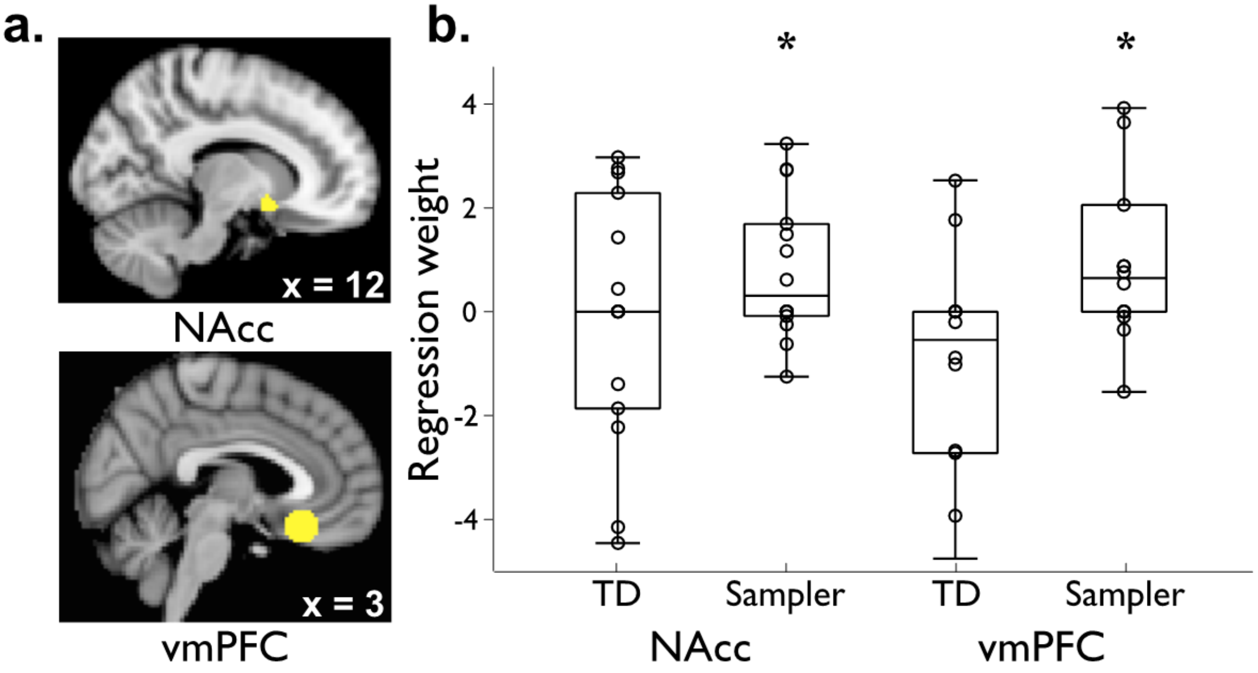

Figure S2: **Sampling model fit to neural decision variables. a. Regions of interest.** We isolated voxels of interest that corresponded to previous reports of the neural substrates for the decision variables analyzed here: Chosen Value (CV), in ventromedial prefrontal cortex (vmPFC), and Reward Prediction Error (RPE), in the nucleus accumbens (NAcc). **b. Simultaneous regression.** Candidate timeseries for each decision variable were generated according to each of the two models, and entered into a simultaneous regression against the BOLD timeseries extracted from the relevant ROI. Each plotted point represents the regression coefficient for the respective model-timeseries pair; box plots display the mean and interquartile range (* $P < 0.05$). Both regressions support the hypothesis that the Sampler model underlies neural signals (NACC-RPE: $t(13) = 2.2134$, $P = 0.0454$; vmPFC-CV: $t(13) = 2.2604$, $P = 0.0416$).

Supplemental Tables

Task	Simulated model	Fraction best-fit	log Bayes
Expt 1	TD	0.815	12.8726 (0.8499)
	Sampler	0.897	8.4295 (0.7734)
Expt 2	TD	0.887	5.9292 (1.5065)
	Sampler	0.910	3.5762 (0.6757)

Table S1: **Confusion matrix for Sampler and TD models.** For each experiment and each model, we simulated 1,000 participants using the given model as ground-truth. Each individual simulated participant used a set of parameters selected at random from the parameters fit to human participants. Both models were then fit to each simulated participant's choices. Shown are the fraction of simulated participants best-fit by the ground-truth model and the mean (SEM) log Bayes factor in favor of that model.

Model	α	α^{evoked}	β	β^c	log Bayes
TD	0.5552 (0.0862)	-	1.7551 (0.6845)	-0.0930 (0.2354)	6.9182 (1.3227)
TD-evoked	0.5269 (0.0842)	0.2135 (0.0545)	2.3351 (0.7223)	-0.0962 (0.2381)	5.9167 (1.2677)
Sampler	0.5393 (0.0583)	0.4386 (0.0990)	2.2869 (0.4943)	0.5855 (0.3215)	-

Table S2: **Fit model parameters for Experiment 2, including the TD-evoked model.** The parameters shown are the mean (SEM) across subjects. The final column shows the mean (SEM) of the log Bayes Factor versus the Sampler model (smaller is better).

α^{TD}	α^{Sample}	β^{c}	β^{TD}	β^{Sample}	α^{evoked}	log Bayes
0.4275 (0.0653)	0.5670 (0.0521)	0.5281 (0.2815)	0.0580 (0.4654)	2.0910 (0.5187)	0.6005 (0.0871)	0.9700 (1.1015)

Table S3: **Fit model parameters for the Hybrid model.** The parameters shown are the mean (SEM) across subjects. The final column shows the mean (SEM) of the log Bayes Factor versus the Sampler model.

Supplemental notes

Simulated model fits

To demonstrate that these models are, in fact, distinguishable, we simulated the models each running 1,000 instantiations of each experiment, each instance with separately initialized payoff and outcome timeseries. Each model simulation was run using parameters as fit to one randomly drawn subject from the respective Experiment. The Sampler model drew one sample before each choice. For Experiment 2, simulated subjects responded to memory probes correctly the same percentage as did our real subjects. We then fit both models to each population of 1,000 simulated subjects. The result of these fits is shown in Table S1.

For Experiment 1, subjects simulated using the TD model, 81.5% were best-fit by the TD model, by an average log Bayes factor of 12.8726 (SEM 0.8499). For Experiment 1, subjects simulated using the Sampler model, 89.7% were best-fit by the Sampler model, by an average log Bayes factor of 8.4295 (SEM 0.7734).

For Experiment 2, subjects simulated using the TD model, 88.7% were best-fit by the TD model, by an average log Bayes factor of 5.9292 (SEM 1.5065). For Experiment 2, subjects simulated using the Sampler model, 91.3% were best-fit by the Sampler model, by an average log Bayes factor of 3.5762 (SEM 0.6757).

In both datasets, the corresponding simulated model was a superior fit, for the bulk of the population and on average at the individual level.

Simulated regression results

We show that the regression results follow from the episodic sampling model. To simulate the model, we generated 50 populations of 20 simulated subjects, each of whom ran a unique instantiation of the task (with different payoff timeseries and outcomes), and fit the regression model to each population. Simulated subjects drew one sample before each decision, used the

mean choice parameters as fit to the human population, and gave, on average, accurate responses to memory probes at the same rate as did real subjects. Figure S1 shows the average regression weights, across these populations, for each variable of interest.

Alternative forms of choice noise

One potential explanation for the superior fit of the sampling model is that it simply captures additional stochasticity in subjects' choices, over and above that captured by the standard softmax choice function. For instance, subjects could, with some probability ε, select the highest valued option, rather than selecting based on the difference in value between the two options ([52]; Equation S1).

$$p_t(a = A_i) = \epsilon\left(Q_{t-1}^{TD}(A_i) = \max\left(Q_{t-1}^{TD}(\cdot)\right)\right) + (1 - \epsilon)\left(\frac{e^{\beta^C I_t^C + \beta^{TD} Q_t^{TD}(A)}}{\sum_j e^{\beta^C I_t^C + \beta^{TD} Q_t^{TD}(a_j)}}\right) \quad \text{(S1)}$$

However, model comparison did not provide evidence in favor of this "ε-greedy" approach. In Experiment 1 the Sampler model was favored for 15/20 subjects, by a mean Bayes Factor of 3.0042 (SEM 1.8137, exceedance probability > 0.99), while in Experiment 2 the Sampler was favored for 19/21 subjects, by a mean Bayes Factor of 6.734 (SEM 1.7078, exceedance probability > 0.99).

Neuroimaging reanalysis

Given the Sampler's superior fit to behavior, we used the neuroimaging data collected alongside Experiment 1 [4] to ask whether the expectation and learning variables predicted by the sampling model could provide a better explanation of BOLD signal than did the corresponding variables extracted from a TD model. Specifically, we tested whether the well-studied neural correlates of key decision variables–Chosen Value (CV) and Reward Prediction Error (RPE)–were better predicted by the sampling model than by TD. We first identified regions of interest (ROIs) encompassing areas previously shown to reflect this activity: ventromedial prefrontal cortex / medial orbitofrontal cortex (hereafter: vmPFC) for chosen value, and Nucleus Accumbens (NAcc) for RPE (Figure S2a). Extracting the timeseries of activity within these ROIs, we next performed a simultaneous regression containing the timeseries of variables predicted by both models, along with several regressors of no interest. Comparing the distribution of resulting per-participant regression weights against zero using a two-tailed, one-sample t-test, we evaluated whether each model was a significant predictor of the target BOLD timeseries.

The regressors were slightly, but reliably, correlated between models (for RPE: mean R = 0.1093, P = 0.0215; for CV: mean R = 0.2701, P = 0.0002). To test the exclusive contribution of the Sampler-derived predictor variables, we orthogonalized the RPE and CV timeseries as generated using the Sampler against their TD counterparts, and entered each set of TD and Sampler predictors into a simultaneous regression on the BOLD timeseries.

In both cases, the predictions of the Sampler model captured additional variance in the BOLD

timeseries that was not modeled by TD (Figure S2b). Across participants, regression on the NAcc timeseries revealed significant contribution of the RPE variable as generated by the Sampler model ($t(13) = 2.2134$, $P = 0.0454$), but not TD ($t(13) = -0.1614$, $P = 0.8742$). Similarly, the Chosen Value regressor generated by the Sampler model was a significant predictor of the vmPFC timeseries ($t(13) = 2.2604$, $P = 0.0416$), while the TD version was not ($t(13) = -1.0835$, $P = 0.2983$).

Adding evoked trials to the TD model

We augmented the TD model to incorporate rewards from bandit trials evoked by valid memory probes. Specifically, we added an additional parameter, α^{evoked}, for a new, augmented TD update applied to rewards r_i^{evoked}during memory probe trials (Equation S2). This parameter allowed the weight given to evoked bandit outcomes to vary, reflecting the fact that the sampling mechanism may itself be stochastic in nature — not every probe trial will successfully trigger a recall of the associated context, even those on which participants exhibit correct recognition memory.

$$Q_t^{TD}(a) = Q_{t-1}^{TD}(a) + \alpha^{evoked}(r_i^{evoked} - Q_{t-1}^{TD}(a)) \qquad \text{(S2)}$$

Table S2 expands the comparison from the main text to include this TD-evoked model. After correcting for the additional parameter, the model was a slightly better fit to participants' behavior than the plain TD model. It was not, however, a superior fit to the Sampler model.

Hybrid Sampler and TD model

The evidence across several studies shows that multiple valuation systems contribute to choices, either simultaneously or across time [6,12,35]. The current study provides evidence that one component of this valuation architecture involves evaluating samples from episodic memory. (Of course, we do not know to what extent this influence is coextensive with, e.g. model-based learning as otherwise defined.)

To test the possibility that such a "hybrid" model could account for choices in this task, we implemented a hybrid model combining the TD and episodic sampling models and tested it on Experiment 2. The model has six parameters: a learning rate for the TD component α^{TD}, a decay rate for the Sampler component α^{sample}, a choice stickiness term β^{c}, a softmax temperature for the TD value β^{TD}, a softmax temperature for the β^{Sample}, and a decay rate for evoked trials α^{evoked}. Choice probability is computed using Q-values derived from each model, as specified in the main text, and taken over all possible combinations of samples (following Equation 5 in the main text).

Parameter estimates from this model show that that most of the weight is on the sampler model (in the sense that it has a much higher softmax temperature, i.e. its values have a larger effect on choice – indeed, the weight on the TD model is not reliably different from zero). Accordingly, the addition of a TD component to the sampler model was not robustly justified in light of the

additional free parameters. On average, across subjects, log Bayes Factors were mildly in favor of the Sampler model (mean 0.9700, SEM 1.1015, exceedance probability 0.8999), and the Sampler was a better fit for 13 out of 21 subjects individually. The fit parameters and model comparison results are shown in Table S3.

In sum, after accounting for the additional free parameters, this hybrid model was not a clearly superior fit to behavior than the episodic sampling model taken alone. However, we think that–in an experiment designed to distinguish these two possibilities–a more sophisticated architecture (perhaps employing a common cached value representation as in DYNA [36]) could possibly prove a superior explanation of behavior.

References

. [53] Hanan Shteingart, Tal Neiman, and Yonatan Loewenstein. The Role of First Impression in Operant Learning. Journal of Experimental Psychology: General, 142(2):476–488, Aug 2012. ISSN 1939-2222. doi: 10.1037/a0029550. URL http://www.ncbi.nlm.nih.gov/pubmed/22924882.
